# Temptations of friends: adolescents’ neural and behavioral responses to best friends predict risky behavior

**DOI:** 10.1093/scan/nsy028

**Published:** 2018-04-18

**Authors:** Marigrace Ambrosia, Kristen L Eckstrand, Judith K Morgan, Nicholas B Allen, Neil P Jones, Lisa Sheeber, Jennifer S Silk, Erika E Forbes

**Affiliations:** 1 Department of Psychiatry, University of Pittsburgh, Pittsburgh, PA 15213, USA; 2 Department of Psychology, University of Oregon, Eugene, OR, USA; 3 Oregon Research Institute, Eugene, OR, USA; 4 Department of Psychology; 5 Department of Pediatrics; 6 Center for the Neural Bases of Cognition, University of Pittsburgh, Pittsburgh, PA 15213, USA

**Keywords:** reward, brain, risky behavior, adolescent development, social

## Abstract

Adolescents are notorious for engaging in risky, reward-motivated behavior, and this behavior occurs most often in response to social reward, typically in the form of peer contexts involving intense positive affect. A combination of greater neural and behavioral sensitivity to peer positive affect may characterize adolescents who are especially likely to engage in risky behaviors. To test this hypothesis, we examined 50 adolescents’ reciprocal positive affect and neural response to a personally relevant, ecologically valid pleasant stimulus: positive affect expressed by their best friend during a conversation about past and future rewarding mutual experiences. Participants were typically developing community adolescents (age 14–18 years, 48.6% female), and risky behavior was defined as a factor including domains such as substance use, sexual behavior and suicidality. Adolescents who engaged in more real-life risk-taking behavior exhibited either a combination of high reciprocal positive affect behavior and high response in the left ventrolateral prefrontal cortex—a region associated with impulsive sensation-seeking—or the opposite combination. Behavioral and neural sensitivity to peer influence could combine to contribute to pathways from peer influence to risky behavior, with implications for healthy development.

## Introduction

Adolescents are notorious for engaging in risky behavior (Dahl, 2004; Steinberg, 2008). Driven by high levels of sensation seeking (Steinberg *et al.*, 2008), adolescents are more likely than adults or children to seek high-intensity rewarding experiences that have potential consequences for their health and safety. These include engaging in normative thrill-seeking behaviors such as dangerous driving, sexual intercourse without condom use and drug use, as well as foregoing more preventative behaviors that could promote health and safety, such as the use of seat belts or bicycle helmets (CDC, 2010).

Peer social context is a key factor in adolescents’ risk-taking: Risky behaviors such as reckless driving, substance use, and criminal activity are most likely to occur while adolescents are in the presence of peers (Albert *et al.*, 2013). Adolescence is also a developmental period of substantial changes in social context and social behavior, with the emergence of romantic and sexual relationships, the development of intimate friendships and the enhanced salience of status among peers (Choukas-Bradley *et al.*, 2015). Conceptual models of adolescent development emphasize that a central force influencing changes in behavior, affect and physiology is *social reorientation*, whereby the behavioral and neurobiological aspects of social-cognitive and affective processes change to prioritize peer relationships, such that friendships, sexual relationships and romantic relationships become increasingly salient (Blakemore and Robbins, 2012; Somerville, 2013). Not surprisingly, peer influence, especially for daring behaviors, becomes a more prominent motivator than parental influence or personal decision-making at this age (Liao *et al.*, 2013).

*Social reward*, especially experiences marked by high positive affect and a peer context, is critical to adolescents’ risky behavior. Indeed, evidence from behavioral and neuroscience research supports adolescents’ intense sensitivity to rewarding and peer contexts. Compared with adults, adolescents take more risks during simulated driving in the presence of peers (Chein *et al.*, 2011), are more easily distracted by rewards during cognitive control tasks (Somerville *et al.*, 2011), and display greater response to pleasant stimuli in reward-critical regions such as ventral striatum (Galván *et al.*, 2006). At an individual differences level, adolescents who are prone to deriving a strong sense of reward from peer relationships may respond to the unique social development experiences of adolescence with more frequent or intense engagement in risky behaviors. With increased value placed on enhancing social status, impressing peers and seeking thrills, adolescents who are more sensitive to their peers’ influence or respond to their peers’ positive affect with more enjoyment could be most liable to risky behavior.

Behaviorally, adolescents’ conversations with friends can be a context for promoting rule-breaking behavior (Dishion *et al.*, 1996). This potentially occurs via the experience of social reward. In particular, variability in *reciprocal positive affect*, or the behavioral tendency to respond to another person’s positive affect with expressions of positive affect, could reveal the sensitivity to social reward that makes some adolescents engage in higher rates of risky behaviors. Because peer relationships have important value for social functioning during adolescence, close friends could provide a behavioral setting for eliciting reciprocal positive affect. Interactions with close friends could also have the potential to elicit variability in neural responses to social reward.

Neurally, individual differences in reward-circuit function are associated with adolescents’ risky behavior (Galván *et al.*, 2007) and susceptibility to peer influence (Pfeifer *et al.*, 2011). Thus, because of the unique developmental link between peer social reward and risky behavior that emerges in adolescence, neural sensitivity to peer reward could serve as a trait-like vulnerability factor for risky behavior during this phase of life. Indeed, recent findings have indicated that left VLPFC response to reward corresponds to traits related to risky behavior (Chase *et al.*, 2017).

Risky behavior could be more likely for adolescents who have sensitive neural systems for processing not just reward, but social reward in particular. Adolescents’ response to social reward, increased social focus, and rates of risky behavior are putatively driven by development in a combination of reward, social and self-regulatory networks (e.g. Casey *et al.*, 2011). The reward network includes the ventral striatum, the primary target of ventral tegmental dopamine neurons that is considered the hub of reward circuitry; the amygdala, which responds to reward receipt; and the medial prefrontal cortex (PFC), which contributes to affective experience and regulation in response to reward (Haber, 2016). The social and self-processing network includes the temporoparietal junction, which is implicated in theory of mind and responds to social reward (e.g. Eckstrand *et al.*, 2017); the medial PFC, which processes both social and self-relevant information; and the posterior cingulate and precuneus, a combined hub of the default-mode network with a role in self-referential, autobiographical and agentic processing (Nelson *et al.*, 2005; Northoff and Hayes, 2011; Blakemore and Mills, 2014). Other regions contribute to multiple networks involved in processing social reward. The ventromedial PFC is postulated to compute reward valuation, affect regulation and social cognition (Delgado *et al.*, 2016); the anterior insula contributes to reward seeking and reward responding but also appears to compensate for social pain (Cristofori *et al.*, 2015) and contribute to adolescents’ risky behavior (Smith *et al.*, 2014); and the ventrolateral prefrontal cortex (VLPFC) is central to several aspects of affect and self-regulation (Braunstein *et al.*, 2017), including impulsive sensation-seeking (Chase *et al.*, 2017). This intersecting set of networks undergoes development during adolescence and coordinates the increasingly sophisticated social-affective processing and corresponding behaviors, including risky behavior, that emerge in adolescence.

This study was guided by the stance that adolescents’ neural response to peer social reward (here, positive affect with a close friend) may differ greatly across adolescents and that such individual differences (i.e. variability across people in magnitude of neural response) may predict individual differences in risky behavior. From developmental psychopathology and clinical neuroscience perspectives, these individual differences could tip the balance toward risky behavior in contexts of peer positive affect. Specifically, we hypothesized that adolescents with a combination of heightened neural response and reciprocal positive affect response to social reward will engage in a higher level of a range of risky behaviors.

To test this hypothesis, we developed a novel fMRI social reward paradigm using dynamic, personally relevant peer stimuli. This paradigm uses stimuli high in positive affect and is individualized based on video from a conversation with a close friend about a shared, high-intensity, pleasant experience. We have used similar approaches successfully in parent–child contexts to assess social-affective responding in an ecologically valid way (Whittle *et al.*, 2009; Morgan *et al.*, 2015). We propose that behavioral response from the conversation can capture reciprocal positive affect, while neural response can capture sensitivity to peer social reward. In all, combining rigorous behavioral observation and functional neuroimaging and using methods based on naturalistic contexts for risky behavior could lead to progress in understanding the social and affective neuroscience of adolescence.

## Materials and methods

### Participants and protocol

Participants were 50 typically developing community adolescents with no history of psychiatric disorder or serious health problems. Participants were ages 14–18 (*M *=* *16.22, s.d.* *=* *1.4), 48.6% female, and 68% European American, 27% African American and 5% mixed race. Of the original 70 participants in the study, 7 did not complete the fMRI scan, due to ineligibility (recent concussion, *n *=* *3; claustrophobia, *n *=* *2; mental health history, *n *=* *2), 3 were unable to be contacted after the initial visit and 2 refused the scan. Of the 58 who completed the entire assessment, 3 did not complete the fMRI task because of technical problems, 1 had inadequate coverage of the ventral striatum (≤80%), 1 had excessive movement (>25% of volumes over 2 mm in any direction) and 3 had missing behavior data because of coding error. All 50 participants had adequate coverage in ventral brain areas and <2 mm movement in any direction. All participants identified a same-gender best friend who attended the lab visit to complete the peer interaction task (*M = *15.82 years, s.d.* = *1.2; race: 38% African American, 62% European American; demographic data on friends were missing for 18 participants).

Participants completed a lab visit with self-report measures and an MRI scan. Most participants (57%) were scanned within 1 month of their lab visit (median* *=* *27.5 days, s.d.* *=* *54.8). The University of Pittsburgh Institutional Review Board approved all research procedures, and written informed consent was obtained from each participant and a parent/guardian.

### Risky behavior

Participants completed the standard high school version of the Youth Risk Behavior Survey [YRBS; Center for Disease Control and Prevention (CDC), 2010], an 89-item self-report instrument developed for epidemiologic research on high school students’ engagement in 6 domains of health-risk behaviors. These are (i) behaviors contributing to injury and violence, (ii) sexual behaviors related to negative outcomes, (iii) alcohol and drug use, (iv) tobacco use, (v) unhealthy dietary behaviors, and (vi) inadequate physical activity. These behaviors are considered risky based on their potential to compromise physical health (e.g. obtaining sexually transmitted infections from intercourse without condom use) or mental health (e.g. developing addiction from frequent use of illicit drugs). Thus, the YRBS does not include reward-seeking behaviors that are adaptive or that have only minor direct consequences for health and wellbeing (e.g. initiating a new romantic relationship, raising one’s hand in class).

Scores for risky behavior were computed based on a single factor created by Youssef *et al.* (2016) from 10 YRBS items selected to represent a broad range of risky behaviors, including substance use, seat belt use, sexual behavior and suicidality. Items in the factor include daily cigarette smoking, seat belt use while riding in a car, number of lifetime sexual partners, and having gotten into a physical fight (see Supplementary Table S1 for all items). The factor was tested with confirmatory factor analysis in a sample of 174 community adolescents and then replicated in a sample of 4135 16-year-old adolescents from the 2009 CDC Youth Risk Behavior Surveillance Survey, with good fit and a unidimensional factor structure in both samples (see Youssef *et al.*, 2016 for factor loadings in both samples). All 10 items in the factor were included in this study, as in previous work (Eckstrand *et al.*, 2017). Raw scores were used in analyses, as they were highly correlated with scores adjusted for factor loadings. Risky behavior scores were normally distributed, with higher scores indicating higher levels of general risky (*M *=* *15.51, s.d.* *=5.57, range = 8-27).

### Reciprocal positive affect behavior

Each adolescent (*target*) and her or his best friend were video recorded as they engaged in a 10-min conversation, with 5 min devoted to ‘the most fun you’ve ever had together’ and 5 min devoted to ‘a fun or exciting event you’d like to plan together.’ Topics were chosen based on participants’ responses to a list of pleasant events, with examples including high school graduation, visiting a local amusement park, soccer camp and weekend parties with alcohol and drug use. Similar tasks have been used successfully in families of healthy (Whittle *et al.*, 2009) and depressed (Sheeber *et al.*, 2007) adolescents.

Affective behavior from the conversation was coded from video for two purposes, in two ways, by separate coding teams: (i) using the LIFE coding system (Hops *et al.*, 1995) to obtain detailed measurement of reciprocal positive affect, which was the main regressor in our models; and (ii) using ratings of participant and friend positive and neutral affect to select segments to create stimuli in the fMRI task (see below). For LIFE coding to obtain reciprocal positive affect, trained observers blind to hypotheses coded adolescents’ affect expression and verbal content from video in real time. Four constructs—aggressive, positive, dysphoric and other—were derived from the individual codes. These constructs are based on the theoretical rationale of the LIFE system, which was developed to examine the function of emotions in social contexts. The positive affect construct, which was the focus of our analyses, captures *happy, caring* and *facilitative behavior* codes.

Reciprocal positive affect—to examine the tendency to respond to a close friend’s positive affect in kind, rather than a general tendency toward positive affective behavior—was then computed from the positive affect construct of LIFE codes. This variable was computed using the Generalized Sequential Querier Program (Bakeman and Quera, 2011). Conditional lagged probabilities for positive affect were computed for participants’ behavior given friends’ behavior, by considering participants’ behaviors occurring from 1 s after the onset until 1 s after the offset of each observation of friends’ positive affect (i.e. instances in which the target participant expressed positive affect during or after the best friend’s expression of positive affect). This resulted in a 2×2 contingency table for each dyad, reflecting the probability of the consequent (participant positive affect) given the antecedent (friend positive affect), as compared to the probability of the consequent given all other peer behavior antecedents and the probability of all other participant affective behaviors given friend positive affect. From these tabled values of joint probability, adjusted residuals were computed for analyses. These values reflected associations greater than expected by chance (i.e. positive values) or lower than expected by chance (i.e. negative values) (*M = *13.70, s.d.* *=* *8.06, range = −2.95 to 33) and were distributed approximately normally, with a mean of 0 and a variance equal to 1. We note that reciprocal positive affect is intended to capture a behavioral tendency during the entire interaction and is not meaningful across smaller time segments.

### Neural response to friend positive affect

#### Best Friend fMRI task

**Fig. 1.**
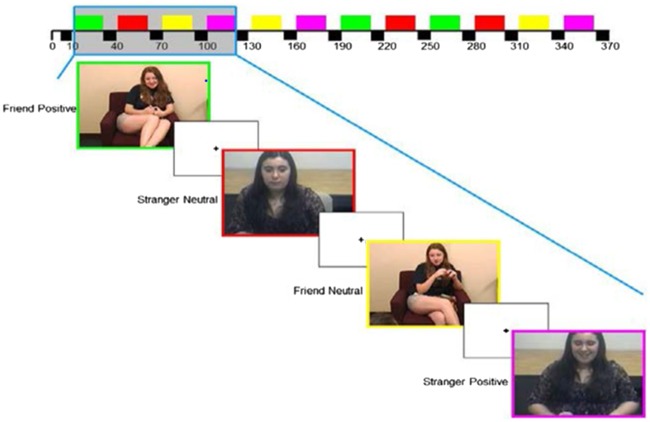
Design of the Best Friend fMRI task, in which adolescents view video clips of positive and neutral affect displays by a same-gender unfamiliar adolescent or by their own same-gender best friend. Video clips from the best friends were drawn from a laboratory-based dyadic interaction in which the adolescents discussed their most pleasant shared experience.

This novel task, personalized for each participant, contained six video clips of the participant’s best friend and six video clips of an unfamiliar, same-gender, control adolescent presented in a block design with 10-s fixation displays between blocks. Blocks were presented in a predetermined, pseudorandom order so that positive and neutral affect blocks alternated and clips with the friend or unfamiliar peer alternated (Figure 1). During the task, participants were instructed to attend to each video and to press a button at the onset of each, to ensure that they were awake and engaged.

Affective behavior from the conversation was coded from video to create task stimuli. Lab conversation videos were coded in 5-s epochs by a team of trained observers using an adaptation of the AFFEX coding system (Izard *et al.*, 1983). This was conducted separately from the coding to compute reciprocal positive affect behavior. Specifically, videos were coded for friends’ positive and neutral affect during the lab conversation (i.e. not target participants’ affect or dyadic affect), and coding data were used to determine each participant’s stimulus segments for the *friend positive* and *friend neutral* conditions of the fMRI task. Positive affect was coded with a score of 0–2 for its presence and intensity and neutral affect was coded with a score of 0 or 1 for its presence only.

Approximately 25% of the videotapes were additionally coded by an extensively trained master coder for reliability (mean ICC for PA = 0.93, range = 0.86–0.96; mean ICC for neutral affect = 0.92; range = 0.89–0.94). The master coder selected 20-s segments based on predominance of positive or neutral affect. As intended, the best-friend positive affect stimuli included higher levels of positive affect than neutral stimuli [(*M *=* *1.55; s.d. = 0.37) and (*M *= 0.08; s.d.=0.15); *t*(232) = −40.24, *P *= 0.000], and best-friend neutral stimuli included higher levels of neutral affect than positive stimuli [(*M *= 0.93; s.d.* *= 0.16) and (*M *= 0.06; s.d.* *= 0.16); *t*(228) = 41.48, *P *= 0.000].

Stimuli included the head and shoulders of the best friend (i.e. not a view of the participant herself) and audio of both adolescents. We made efforts to ensure that video clips were equivalent in lighting, camera angle, zoom and intensity of affect. Stimuli included segments drawn from both past- and future-focused parts of the lab conversation, with positive affect clips tending to be from the past conversation (66%) and neutral clips tending to be from the future conversation (60%). Similar to methods used in Whittle *et al.* (2012) and Morgan *et al.* (2015), and to avoid participants’ inadvertent familiarity with adolescents in the control conditions, stimuli for the *unfamiliar-peer positive affect* and *unfamiliar-peer neutral affect* control conditions were drawn from video segments of dyadic interactions of adolescent actors from Eugene, Oregon. These adolescents’ training allowed them to convincingly portray a conversation with a close friend. Control stimuli were selected using the same procedures used for the best-friend stimuli. As with the best-friend stimuli, the segments selected for the unfamiliar peer positive affect stimuli had higher levels of positive than neutral affect [(*M *= * *1.71; s.d.* *= 0.29) and (*M *= 0.04; s.d.* *= 0.10), respectively; *t*(10) = −13.19, *P *= 0.000], and the segments selected for the unfamiliar peer neutral affect stimuli had higher levels of neutral than positive affect [(*M *= 0.75; s.d.* *= 0.50) and (*M *= 0.06; s.d.* *= 0.16), respectively; *t*(6) = 3.00, *P *= 0.024]. Control stimuli were presented to each participant to match the participant’s gender and approximate age.

Because our focus was positive affect within a familiar peer context (e.g. rather than positive affect from a familiar *vs* unfamiliar peer), the contrast generated for analyses was *friend positive affect *>* friend neutral affect*. This contrast allowed assessment of neural response to a type of social reward that is relevant to adolescents’ risky behavior. The contrast was defined across all three 20-s blocks of friend positive affect stimuli and all three 20-s blocks of friend neutral affect stimuli. The onset and duration of each condition was based on all 20-s segments of best friends’ behavior selected for inclusion as stimuli based on AFFEX coding. Thus, the two conditions had an identical duration of data analyzed.

#### fMRI acquisition and preprocessing

Each participant was scanned using a Siemens 3-T TIM Trio scanner. Structural images were acquired using MPRAGE 160 axial slices, 1.2-mm thick (TR/TE = 2300/2.98 ms, FOV = 256×240 cm^2^, matrix = 256 × 240, flip angle = 9°). BOLD functional images for the friend task were acquired in a single run, with a gradient echo planar imaging sequence and covered 39 axial slices, 3.1-mm thick, beginning at the cerebral vertex and encompassing the entire cerebrum (TR/TE = 2000/28 ms, FOV= 20×20 cm^2^, matrix = 64 × 64, flip angle = 90°).

Preprocessing and analysis of fMRI data were completed using SPM8 (http://www.fil.ion.ucl.ac.uk/spm). Structural images for each participant were segmented to focus on gray matter. For each functional scan, data were realigned to correct for head motion. Volumes with excess motion (>3 s.d. from the subject’s mean, >0.5 mm scan-to-scan translation, or >0.01 degrees of scan-to-scan rotation) were identified using Artifact Detection Toolbox (ART; http://www.nitrc.org/projects/artifact_detect/) software. Preprocessed data were inspected prior to first-level analysis to ensure that all participants had fewer than 25% of volumes with excessive movement detected by ART. Images were then spatially normalized into standard stereotactic space (Montreal Neurological Institute template) using a 12-parameter affine model and smoothed with a 6 mm full-width at half-maximum Gaussian filter. Voxel-wise signal intensities were ratio normalized to the whole-brain global mean. Voxels were resampled during preprocessing to 2 mm^3^.

### Data analyses

First-level tests for effect of task within each participant were calculated at each voxel using paired *t*-tests for friend positive affect > friend neutral affect. In addition, exploratory analyses to address the specificity of effects examined the contrasts unfamiliar-peer positive affect > unfamiliar-peer neutral affect and (friend positive affect > friend neutral affect) > (unfamiliar-peer positive affect > unfamiliar-peer neutral affect) (see below).

Second-level analysis was conducted using a within-sample *t-*test masked for regions that respond to fMRI paradigms focusing on social stimuli. The mask was obtained from the Neurosynth platform (neurosynth.org; Yarkoni *et al.*, 2011), which provides meta-analytic findings across fMRI studies measuring specific constructs. Given our focus on social reward, we selected the search term *social*, which, at the time of analysis, yielded a map of results from exactly 1000 studies of social stimuli. The map included the following regions: dorsomedial, ventromedial and ventrolateral PFC; precuneus; temporoparietal junction (including right superior temporal gyrus); temporal pole; amygdala; and ventral striatum (Supplementary Figure S1). Type I error for the within-sample *t* test was controlled by applying a voxel-wise height threshold of *P *< 0.0001 and family-wise error correction at cluster level of *P *< 0.05, which is consistent with current recommendations for rigorous adjustment for multiple comparisons in fMRI research.

Mean BOLD response for a sphere of 2 mm around the peak voxel of each cluster resulting from the second-level analysis above was then extracted for moderation analyses. One moderation model was computed for each cluster. Additional analyses were performed extracting (i) the BOLD response within each of the significant clusters and (ii) the BOLD response within a priori anatomical masks of social reward regions significantly activated by the task using a small volume correction. Age and gender were included as covariates in *t*-tests to adjust for their potential role, even though participants’ risky behavior, affective behavior, and neural response to reward did not vary with gender, race or age (all *P*s > 0.07).

Moderation analyses tested the hypothesis that behavioral×neural response to peer social reward predicts risky behavior. Analyses were conducted using the PROCESS macro for SPSS (Hayes, 2012). In these analyses, which do not require significant association between independent variable and dependent variable to test moderation, reciprocal positive affect was entered as the independent variable, extracted BOLD variable for each region was entered as the moderating variable, and risky behavior was entered as the dependent variable. Type I error in moderation models was controlled using the sliding linear model (SLIM; Wang *et al.*, 2011), a method designed for data sets with dependence structure, as is the case for fMRI variables extracted from second-level model described above. Age and gender were not covaried in moderation analyses given that they were previously controlled for in the neuroimaging analyses. However, supplementary analyses revealed that the addition of age and gender as covariates did not affect the significance of the results.

## Results

### Neural response to the Best Friend task

**Fig. 2.**
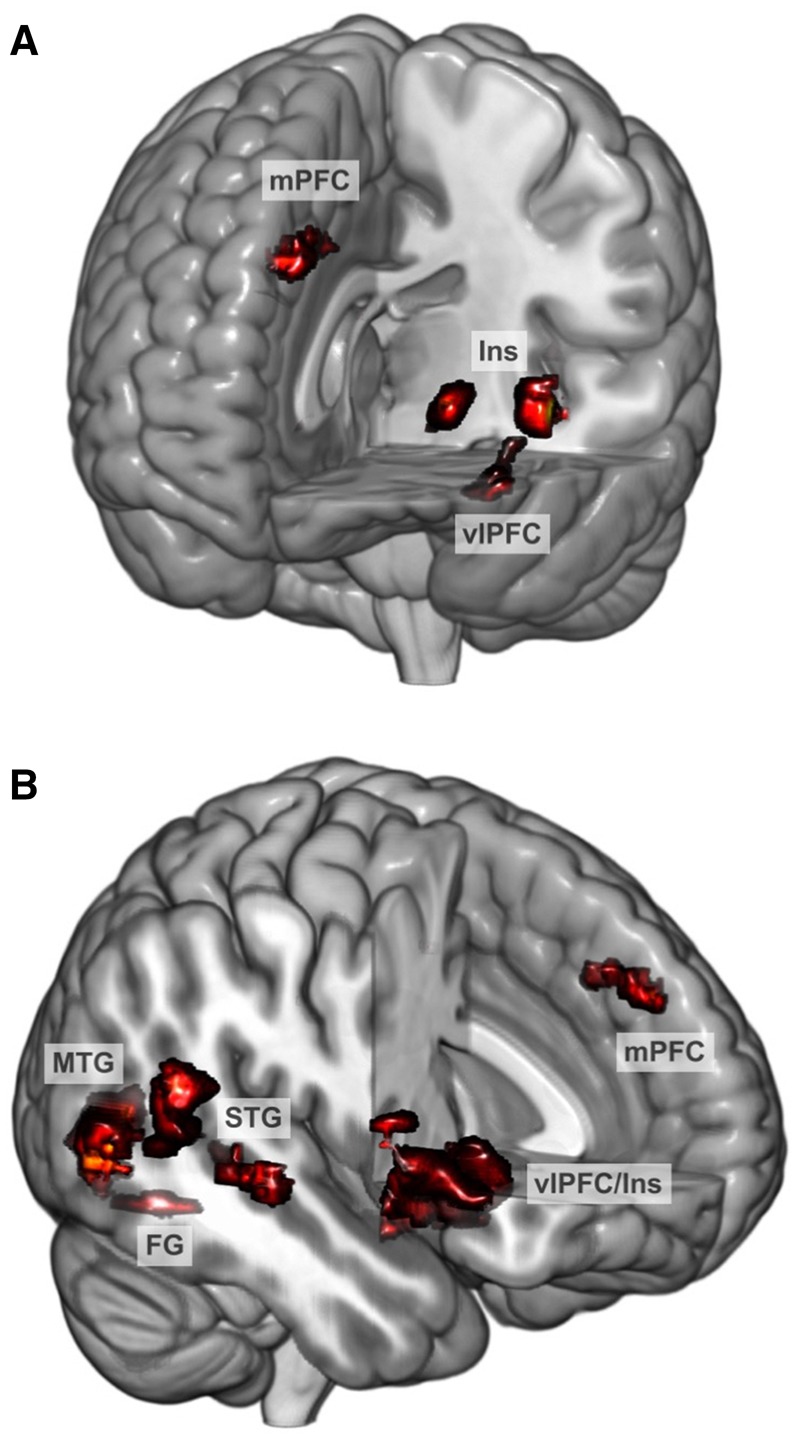
Adolescents’ neural response to videos of their best friends’ positive affect relative to neutral affect, masked for meta-analytic findings on regions activated during social processing.

Participants exhibited neural response to best friend positive affect relative to best friend neutral affect in nine regions that have been reported, across studies, to respond to social stimuli: VLPFC (bilateral), dorsomedial PFC, superior temporal gyrus (bilateral), middle temporal gyrus (bilateral), anterior insula, and fusiform gyrus (Table 1 and Figure 2). Whole-brain analyses confirmed the response of these regions (see Supplementary Table S2 and Supplementary Figure S2). Thus, the task effectively engaged the circuitry of interest.

**Table 1.** Neural response to the Best Friend task in regions associated with social processing

Region	*P*_FWE_	Cluster size	*t*	*x*	*y*	*z*
L VLPFC	<0.001	227	6.89	−54	10	0
R Temporoparietal junction	0.001	156	6.65	56	−20	2
R Middle temporal gyrus	<0.001	391	6.58	48	−62	6
L Anterior insula	0.014	83	5.83	−30	26	4
R VLPFC/insula	<0.001	610	5.76	46	14	2
R Superior temporal gyrus	<0.001	239	5.72	62	−40	22
Dorsomedial prefrontal cortex	0.007	102	5.49	0	50	40
L Middle temporal gyrus	0.023	70	5.44	−60	−56	6
R Fusiform gyrus	0.016	79	5.17	38	−58	−14

Note: Threshold for statistical significance was *P* < 0.0001, with cluster *P*_FWE_ < 0.05. Degrees of freedom = 1, 47. The contrast tested was Friend Positive Affect > Friend Neutral Affect, with masking by Neurosynth meta-analytic results for fMRI studies of social stimuli. Cluster size is presented in voxels. Coordinates (*x, y, z*) are in MNI space and refer to the voxel with the maximum *t*-score in each cluster. L, left; R, right.

Regression analyses in SPM indicated that neural response to best friends’ positive affect was unrelated to reciprocal positive affect behavior or risky behavior. Also, bivariate correlations revealed that reciprocal positive affect and risky behavior were unrelated (*r* = 0.07, *P *= 0.63).

### Interaction of neural response and behavioral response to friend positive affect as a predictor of risky behavior

The moderation model for the left VLPFC cluster was significant [*R*^2^ = 0.23, *F*(3, 45) = 4.43, *P *= 0.008, SLIM *P *= 0.01]. Based on outlier analyses, one case was excluded from this analysis based on having a studentized residual >2. Furthermore, moderation analyses indicated that the combination of left VLPFC response and contingent positive affect behavior predicted risky behavior [Δ*R*^2^ = 0.19, *F*(1, 45) = 11.21, *P *= 0.002, SLIM *P *= 0.03]. That is, adolescents’ left VLPFC response to their best friends’ positive affect moderated the association between their shared positive affect during an interaction with that friend and their behavior across multiple health-risk domains.

Moderation models with the other clusters activated by the task were nonsignificant (*F *= 0.03 for right temporoparietal junction, 1.78 for right middle temporal gyrus, 1.41 for left anterior insula, 1.09 for right VLPFC, 2.46 for right superior temporal gyrus, 0.86 for dorsomedial PFC, 0.01 for left middle temporal gyrus, 0.01 for right fusiform gyrus; *P*s = 0.12–0.94).

**Fig. 3.**
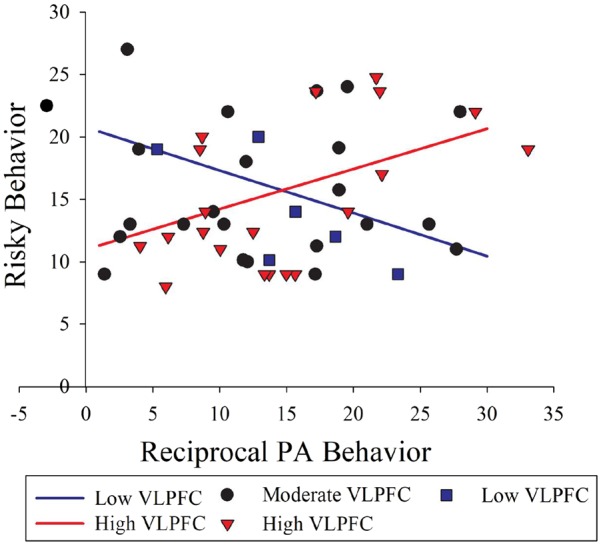
Illustration of conditional effects of adolescents’ contingent positive affect (PA) behavior on their real-life risky behavior at high and low levels of left VLPFC response to the Best Friend Task, which involves peer social reward. The lines’ slopes (−0.07, 0.80) reflect the levels of VLPFC response at which contingent positive affect behavior and risky behavior become negatively and positively correlated, respectively. These illustrate the high and low values beyond which VLPFC is a statistical moderator, with better accuracy than the convention of presenting values reflecting ±1 s.d. of the mean value for the moderator. The larger number of cases for the combination of higher positive affect and higher VLPFC (i.e. higher region of significance; red triangles) than for lower positive affect and lower VLPFC (i.e. lower region of significance; blue circles) likely reflects the combinations evident in a community sample of typically developing adolescents. In other words, having fewer cases in the lower region of significance does not indicate that the interaction effect is driven by outliers. Scatter points represent actual data values. One case was removed because analyses indicated that it was an outlier for VLPFC response.

To further examine moderation findings, we applied the Johnson–Neyman technique, which explicates an interaction effect involving a continuous moderator variable by indicating *regions of significance*, or the values of a continuous moderator variable above or below which there is a conditional association between the independent and dependent variables (Preacher *et al.*, 2006). This technique indicated that left VLPFC response moderated the association between reciprocal positive affect behavior and risky behavior, with significant interaction effects evident at both low and high levels of neural response [conditional effects at VLPFC response <−0.07 (12% of cases; *n *=* *8) or >0.80 (59%; *n *=* *20); Figure 3]. That is, adolescents who reported more behaviors such as using illicit substances, getting in fights or having multiple sexual partners were those with either higher left VLPFC response and higher reciprocal positive affect (the predicted combination of effects) *or* lower left VLPFC response and lower reciprocal positive affect (an unexpected combination of effects). Other regions that exhibited response to a best friend’s positive affect—such as dorsomedial PFC and temporoparietal junction—did not significantly moderate the association between reciprocal positive affect and risky behavior.

### Exploratory analyses

We conducted two sets of additional analyses to examine the results further. First, to test the specificity of findings to the best-friend context, we used a within-sample *t* test of response to *unfamiliar-peer positive affect > unfamiliar-peer neutral affect* and a paired *t* test of response to (*friend positive affect > friend neutral affect*) *vs* (*unfamiliar-peer positive affect > unfamiliar-peer neutral affect*). No significant clusters emerged for response to unfamiliar peer positive affect or for unfamiliar peer > best friend positive affect. However, adolescents exhibited greater response to best friends’ positive affect than to unfamiliar peers’ positive affect in a cluster including the VLPFC, superior temporal gyrus, and inferior frontal gyrus [119 voxels, *t *=* *5.78, *P *< 0.001, cluster *pFWE *= 0.01, (−54, 10, 2)]. Second, to test the potential contribution of ventral striatum, which did not emerge at our specified statistical threshold but which increases in adolescence and is related to reward sensitivity (e.g. Braams *et al.*, 2015), we probed results using a lower statistical threshold of *P *< 0.005. No significant clusters emerged in the ventral striatum, and the only striatal area showing response at this more liberal threshold was the caudate tail. We also used an anatomical ventral striatum mask for the direct comparison of best friend and unfamiliar peer positive affect described above, and no significant clusters emerged.

## Discussion

Adolescents’ combined neural response and behavioral response to their best friends’ PA—but, tellingly, neither type of response alone—was associated with their engagement in a range of real-world risky behaviors. Surprisingly, greater engagement in risky behaviors was associated with the combination of higher neural and higher behavioral response *and* the combination of lower neural and lower behavioral response to best friends’ positive affect. The neural response moderating the association between positive affect behavior and risky behavior was evident in the left VLPFC, a region associated with trait-like tendencies toward impulsive sensation-seeking.

Both higher and lower reciprocal positive affect were related to higher levels of risky behavior. Moreover, this was specifically the case for those with higher and lower VLPFC response, respectively. This seeming u-shaped association between neural–behavioral response to friend positive affect and risky behavior suggests two possible pathways to adolescents’ risky behavior: high susceptibility to social reward and intense positive affect, or relative indifference to social reward. Perhaps strong reactivity to social reward promotes thrill-seeking attempts to enhance or maintain positive affect, whereas weak reactivity promotes engagement in risky activities for other reasons. Adolescents in the former pathway could find themselves with poor health outcomes, but they might also derive some benefits from their tendencies: in the context of typical development, sensitivity to social rewards could predispose adolescents to obtain social status or to engage in adaptive, pro-social behaviors (see Telzer, 2016). Adolescents in the latter pathway might be those with low baseline levels of reward responding or high susceptibility to boredom, for whom risky behaviors serve to compensate for a tendency toward blunted responding (Zuckerman, 1996).

The pathway involving less sensitivity to social reward occurred less frequently in our sample than the pathway involving greater sensitivity. This pathway could be more evident in clinical populations, such as adolescents with serious conduct problems and callous–unemotional traits (Blair *et al.*, 2014); depression, a disorder accompanied by low response to reward in the striatum (e.g. Forbes *et al.*, 2009); or suicidality, a class of risky behavior included in our outcome variable and associated with other risky behaviors (Stewart *et al.*, 2017). Alternatively, we might not have sampled that extreme range of responding. In all, there could be a sweet spot for sensitivity to social reward, whereby extremely low or high intensity of sensitivity could lead to problem-level risky behaviors while moderate intensity leads to levels appropriate for promoting affiliation with peers, adaptive status-seeking and individuation from parents.

Neural and behavioral responses were assessed during an ecologically valid context: a conversation about an intensely positive, shared experience. This allowed us to extend the investigation of adolescents’ risky behavior beyond traditional models of susceptibility to peer influence and measures of isolated, individual responses elicited by standardized, static stimuli. In contrast to most studies of adolescent social processing, which have focused on the mere presence of peers, evaluations by virtual peers, or cognition about peers (e.g. Jarcho *et al.*, 2015; Bolling *et al.*, 2016; Will *et al.*, 2016; see Pfeifer and Blakemore, 2012 for a review), we focused on what is potentially the most powerful, salient context for adolescents’ risky behavior: a dynamic interaction with a close friend involving heightened positive affect. This was also our first investigation employing the innovative Best Friend fMRI task. Building on other recent work using stimuli from family relationships (Whittle *et al.*, 2012; Morgan *et al.*, 2015), the task engaged regions commonly related to social processing, including dorsomedial PFC, ventrolateral PFC, anterior insula and temporoparietal junction. Notably, neural response to positive affect was stronger in the context of close friendship than in the context of a generic peer: a set of regions involved in social and affective processing exhibited more response to the best friend, whereas no clusters exhibited greater response to the unfamiliar peer.

In addition, while extant studies have rarely focused on individual differences, our task examined variability in neural response as a statistical predictor of risky behavior. The stimuli convey the social context, autobiographical history and meaningful pleasant experiences that accompany real-world peer influence on adolescents’ risky behavior. Thus, it appears that dynamic positive affect experienced with a close friend can powerfully engage neural social-affective circuitry and, in combination with positive affect behavior, reveal individual differences in the potentially problematic reward-driven behavior that peaks at adolescence (Dahl, 2004).

Reciprocal positive affect with a close friend predicted risky behavior only in combination with left VLPFC response to the friend’s affect, indicating that positive affect might be especially meaningful in the context of sensitivity in associated neural systems. The left VLPFC, while not a direct focus of our hypotheses, is an intriguing player in affective, reward, and self-relevant processing. Recent work has linked individual differences in the function of left VLPFC—in a subregion similar to that identified in this study—to trait impulsive sensation seeking (Chase *et al.*, 2017) and adolescents’ rule breaking behaviors (Bebko *et al.*, 2014). Left VLPFC is also a putative biomarker of bipolar disorder, a form of mental illness notable for excessive reward-driven behavior (Phillips and Swartz, 2014). This region also plays a role in risky choices (Eshel *et al.*, 2007) and effortful affect regulation (Braunstein *et al.*, 2017; Phillips *et al.*, 2008). Also, while interpretations of BOLD response as trait-like should be undertaken with caution given limited test-retest reliability, the region of left VLPFC in which we observed results is emerging across studies as an indicator of stable tendencies toward sensation-seeking. Left VLPFC involvement could perhaps contribute to adolescents’ risky behavior through the necessity of integrating reward, social, and self-relevant information when modulating the pursuit of rewarding goals.

Contrary to previous findings on the neural correlates of adolescents’ risky behavior (e.g. Braams *et al.*, 2015), we did not observe ventral striatum response. This was the case even when we applied a more liberal statistical threshold. While the ventral striatum plays a central role in basic reward responding and reward learning (Haber, 2016), it might not be as critically involved in responding to complex reward stimuli that also require social and self-processing. In addition, the ventral striatum appears to play an important role in learning (or prediction error) that involves social reward contingencies (Lockwood *et al.*, 2016; Will *et al.*, 2017). Given that our task was designed to assess neural response to social feedback without creating contingencies that might be violated by such feedback, it likely did not engage the ventral striatum in this way. Our exploratory analyses also found that ventral striatum response did not differ between the best-friend positive affect condition and the unfamiliar-peer positive affect condition, suggesting that familiarity might not be sufficient to elicit response.

Similarly, our fMRI task did not elicit response in some other regions putatively involved in social reward (e.g. VMPFC). Response in some expected regions also did not moderate the association between positive affect and risky behavior (e.g. temporoparietal junction). Perhaps these regions contribute to risky behavior but do not serve as mechanisms of the association between sensitivity to peer social reward and engagement in activities such as drug use, physical fights or inconsistent condom use.

Several methodological issues are worth noting. First, our outcome variable was a cross-domain composite of risky behavior (Youssef *et al.*, 2016) measured through self-report. Including real-time, objective measures of risky behavior in natural environments will be valuable in future studies. Second, our coding system yielded a general construct for positive affect, but facets of positive affect such as affection, happiness, excitement and contentment may function differentially in adolescents’ peer social interactions. Third, we did not assess friendship quality and thus were not able to incorporate it into analyses of brain–behavior associations. Fourth, positive fMRI stimuli tended to be segments from the past-focused conversation, whereas neutral stimuli tended to be segments from the future-focused conversation. This was unintended but could reflect the power of real experiences over imagined experiences to elicit positive affect. Fifth, our fMRI paradigm allows a potential role for memory in the best-friend conditions but not the unfamiliar-peer conditions, as participants only took part in the best-friend conversation and actors performed in the unfamiliar-peer conversation. Our analyses are likely not influenced by this difference as they focused on best-friend conditions, but future versions of this paradigm could include an alternative control condition with interactions between the participant and an unfamiliar peer. Finally, risky behavior was defined by potential harm to health or safety, rather than as general impulsivity or adaptive reward-seeking, either of which could have a different pattern of association with neural and behavioral response to best friends’ affect.

In all, this study points to the value of examining brain–behavior interactions when investigating behaviors relevant to adolescents’ mental and physical health. Next steps will include extending this research to larger samples, clinical samples, peer groups as well as dyads and real-time risky behavior. A key, related developmental question is whether associations between peer reward and risky behavior are specific to adolescence. Our findings also represent a methodological advance in assessing adolescents’ affective processing and underscore the importance of ecologically valid, personally relevant paradigms for studying this developmental period.

## Supplementary Material

Supplementary Data
