# Supplementary material for: Temptations of friends: adolescents’ neural and behavioral responses to best friends predict risky behavior
Source: Soc Cogn Affect Neurosci. 2018 Apr 18;13(5):483–91. doi: 10.1093/scan/nsy028 (PMC6007330; doi:10.1093/scan/nsy028)
Supplement: Supplementary Data [file nsy028_scan-17-277-file005.docx]

**Supplementary Table 1.** Youth Risk Behavior Survey Items Included in the Outcome Variable for Risky Behavior

| **Domain** | **Item** |
| --- | --- |
| Physical Safety | - When you rode a bicycle during the past 12 months, how often did you wear a helmet? - How often do you wear a seatbelt when riding in a car driven by someone else? - During the past 12 months how many times were you in a physical fight? |
| Suicide | - During the past 12 months, did you make a plan about how you would commit suicide? |
| Substance Use | - During the past 30 days, on how many days did you smoke cigarettes? - During the last 12 months, has anyone offered, sold, or given you an illegal drug on school property? - During your life, on how many days have you had at least one drink of alcohol? - During your life how many times have you used marijuana? |
| Sexual Behavior | - During your life, with how many people have you had sexual intercourse? - The last time you had sexual intercourse; did you or your partner use a condom? |

*Note*: Youth Risk Behavior Survey (Centers for Disease Control & Prevention, 2010)

**Supplementary Table 2.** Adolescents’ Neural Response to Friend Positive Affect > Friend Neutral Affect

| **Region** | **Cluster *p*_FWE_** | **Cluster Size** | ***t*** | **x** | **y** | **z** |
| --- | --- | --- | --- | --- | --- | --- |
| R Superior Temporal Gyrus, Middle Temporal Gyrus, Insula, VLPFC, Putamen, Temporoparietal Junction, Ventrolateral Prefrontal Cortex | <0.001 | 4200 | 7.52 | 48 | -22 | 8 |
| L Superior Temporal Gyrus, Insula, Ventrolateral Prefrontal Cortex | <0.001 | 1562 | 6.96 | -52 | 10 | 0 |
| L Insula | 0.004 | 121 | 5.83 | -30 | 26 | 4 |
| Dorsomedial Prefrontal Cortex | <0.001 | 189 | 5.74 | -8 | 32 | 42 |
| L Middle Temporal Gyrus | 0.001 | 116 | 5.44 | -60 | -56 | 6 |
| R Fusiform Gyrus | 0.005 | 115 | 5.17 | 38 | -58 | -14 |
| L Middle Occipital Gyrus | 0.001 | 165 | 5.07 | 28 | -82 | 4 |
| L Frontopolar Cortex | 0.006 | 109 | 4.93 | -38 | 48 | 24 |
| L Middle Occipital Gyrus | 0.001 | 185 | 4.79 | -10 | -98 | -2 |
| R Precuneus | 0.005 | 110 | 4.77 | 28 | -64 | 28 |
| R Premotor Cortex | 0.035 | 60 | 5.31 | 58 | -6 | 36 |
| Precuneus | 0.013 | 85 | 5.08 | 0 | -76 | 50 |

*Note*: Whole-brain threshold for statistical significance was *p* < .00005, with cluster *p*_FWE_ < .05. Degrees of freedom = 1, 47. The contrast tested was Friend Positive Affect > Friend Neutral Affect. Cluster size is presented in voxels. Coordinates (x, y, z) are in MNI space and refer to the voxel with the maximum *t­*-score in each cluster. L: left; R: right.

**Supplementary Figure Captions**

**Supplementary Figure 1.** Mask applied to response to the Best Friend fMRI task, as downloaded from the Neurosynth repository of meta-analytic findings in fMRI research (neurosynth.org). This mask indicates findings across exactly 1000 fMRI studies that examined neural response to “social” stimuli. This map was obtained by searching for the key word “social.”

**Supplementary Figure 2.** Results of whole-brain analysis of neural response to positive affect in the context of a close friendship in the Best Friend fMRI task (contrast: Friend Positive Affect > Friend Neutral Affect). The statistical threshold was *p* < .00005, with cluster *p*_FWE_ < .05. This whole-brain analysis yielded findings similar to those in the main analysis, which was masked by meta-analytic findings of neural response to social stimuli (e.g., bilateral ventrolateral prefrontal cortex, anterior insula, superior temporal gyrus).
